# The Effect of Daily Co-Trimoxazole Prophylaxis on Natural Development of Antibody-Mediated Immunity against *P*. *falciparum* Malaria Infection in HIV-Exposed Uninfected Malawian Children

**DOI:** 10.1371/journal.pone.0121643

**Published:** 2015-03-25

**Authors:** Herbert Longwe, Kondwani C. Jambo, Kamija S. Phiri, Nyanyiwe Mbeye, Thandile Gondwe, Tom Hall, Kevin K. A. Tetteh, Chris Drakeley, Wilson L. Mandala

**Affiliations:** 1 Department of Basic Medical Sciences, College of Medicine, University of Malawi, Blantyre, Malawi; 2 Malawi-Liverpool-Wellcome Trust Clinical Research Programme, Blantyre, Malawi; 3 Tropical Haematology Research Unit, College of Medicine, University of Malawi, Blantyre, Malawi; 4 Department of Public Health, College of Medicine, University of Malawi, Blantyre, Malawi; 5 London School of Hygiene and Tropical Medicine, London, United Kingdom; Institut de Recherche pour le Développement, FRANCE

## Abstract

**Background and Objectives:**

Co-trimoxazole prophylaxis, currently recommended in HIV-exposed, uninfected (HEU) children as protection against opportunistic infections, also has some anti-malarial efficacy. We determined whether daily co-trimoxazole prophylaxis affects the natural development of antibody-mediated immunity to blood-stage *Plasmodium falciparum* malaria infection.

**Methods:**

Using an enzyme-linked immunosorbent assay, we measured antibodies to 8*Plasmodium falciparum* antigens (AMA-1, MSP-1_19_, MSP-3, *Pf*SE, EBA-175RII, GLURP R0, GLURP R2 and CSP) in serum samples from 33 HEU children and 31 HIV-unexposed, uninfected (HUU) children, collected at 6, 12 and 18 months of age.

**Results:**

Compared to HIV-uninfected children, HEU children had significantly lower levels of specific IgG against AMA-1 at 6 months (*p* = 0.001), MSP-1_19_ at 12 months (*p* = 0.041) and *Pf*SE at 6 months (*p* = 0.038), 12 months (*p* = 0.0012) and 18 months (*p* = 0.0097). No differences in the IgG antibody responses against the rest of the antigens were observed between the two groups at all time points. The breadth of specificity of IgG response was reduced in HEU children compared to HUU children during the follow up period.

**Conclusions:**

Co-trimoxazole prophylaxis seems to reduce IgG antibody responses to *P*. *falciparum* blood stage antigens, which could be as a result of a reduction in exposure of those children under this regime. Although antibody responses were regarded as markers of exposure in this study, further studies are required to establish whether these responses are correlated in any way to clinical immunity to malaria.

## Introduction

*Plasmodium falciparum* malaria infection is still one of the main causes of under-five child morbidity and mortality in Sub-Saharan Africa [1]. In 2010, the World Health Organisation (WHO) estimated that there were approximately 216 million episodes of malaria worldwide with 174 million cases occurring in sub Saharan Africa, which resulted in over 660,000 deaths mainly in children under-five [2]. Several factors are thought to be responsible for this high mortality rate [3], with the host immune response to the infection playing a crucial role [4].The immune response mechanisms are both antibody and cell mediated [5].

Passive transfer studies from adults who are immune to malaria have shown that naturally acquired antibodies to *P*. *falciparum* are protective against malaria infection [6,7]. In children, whilst immunity against the severe clinical symptoms may be achieved quite early in life [8], immunity to parasite multiplication and growth develops slowly and is dependent on repeated exposure to malaria infections over several years [9]. Exposure to *P*. *falciparum* antigens in children is therefore essential for the acquisition of an effective antibody immune response and substantial disruption of this exposure may delay the development of this humoral immunity against malaria. Antibodies are surrogates of exposure to *P*. *falciparum* and henceforth acquisition of effective immune responses [10]. Antibodies directed against various parasite proteins are thought to have a strong inhibitory effect on parasite invasion of red blood cells [10,11]. Naturally acquired IgG responses to several of the *P*. *falciparum* antigens have been shown to be associated with reduced incidence of malaria [12–15].

Co-trimoxazole (CTX), due to its antibiotic efficacy, is recommended for prophylactic treatment in HIV-infected children having been proven to reduce child mortality and morbidity in this group [16–19]. Similarly, for HIV-exposed but not infected (HEU) children (children born to mothers living with HIV) CTX prophylaxis is recommended from 6 weeks of age until the age at which HIV infection is ruled out and breastfeeding is stopped [19]. Besides having potent antibiotic properties, CTX is also known to be an effective anti-malarial drug [20,21]. Previous studies on malaria chemoprophylaxis using drugs primarily meant to treat malaria have shown that continuous provision of such prophylaxis to young children impairs the development of the host’s natural immunity against malaria thereby increasing the child’s susceptibility to malaria when the intervention is stopped [22–24]. Although CTX is not prescribed to HEU children as a malaria chemoprophylaxis, its anti-malarial effects could have a similar effect on the development of natural immunity.

We conducted this study to investigate the effects of daily CTX prophylaxis on the magnitude and breadth of IgG antibody responses against *P*. *falciparum* blood stage antigens in HEU children. With the assumption that *P*. *falciparum* transmission is heterogeneous even within small geographical areas [25], HIV-unexposed uninfected (HUU) children from the same communities of HEU children were recruited as controls.

## Materials and Methods

### Study site, participants and design

The study was conducted in Zomba district, in the southern region of Malawi. *P*. *falciparum* malaria transmission is stable in this district with an increase in infections during the rainy season from November to April. We recruited HEU children and HUU children from 6 months of age and followed them up until they were 18 months old.

HEU children who had confirmed PCR negative resultswere randomly selected from the ART clinic at the Zomba central hospital. The children received daily CTX prophylaxis beginning at 6 weeks of age until at 12 months of age where we conducted a rapid test to rule out HIV infection and the mothers were asked to stop breastfeeding. The children were eligible for inclusion if there were otherwise healthy, breastfeeding, not under any medication and if the mothers indicated their intention to reside in the catchment area for the duration of the study.

Children were seen every six months and at each visit, 1 ml of venous blood was collected in clot activator tubes, serum separated and stored at -80°C for serological analyses. A further1 ml of venous blood was collected in EDTA tubes for full blood counts. Participants were defined as having uncomplicated malaria when they were febrile at the time of recruitment or a reported history of fever in the past 48 hours or had a positive Rapid Diagnostic Test (RDT) or positive malaria thick and thin slides but had a Blantyre Coma Score of 5 and a haemoglobin concentration above 5g/dL. Asymptomatic carriers of *P*. *falciparum* were defined as thick blood smear negative but RT PCR positive. The study was reviewed and approved by the College of Medicine Research Ethics Committee (COMREC) (P.05/10/954). Individual written informed consent was obtained from the parents or guardians of all the children who participated in the study.

### Detection of *P*. *falciparum* parasites

Presence of *P*. *falciparum* parasites from the peripheral blood of the children during follow up was detected by conventional microscopy and real-time PCR from blood smears and dried blood spots respectively. Briefly, Giemsa-stained thick blood films were prepared and two-experienced microscopists interpreted each slide. Thick smears were deemed negative if 200 high power fields were absent of any *P*. *falciparum* asexual parasites.

Two dried blood spots on Whatman FTA filter paper (WhatmanPlc, Maidstone, UK) were cut from each filter paper by sterilized scissors and placed into 1.5ml tubes per sample. Genomic DNA was then extracted from the blood spots using QIAamp DNA Mini Kit protocol for isolation of DNA from dried blood spot (Qiagen, Germany). Genomic DNA samples were amplified in an assay targeting the *P*. *falciparum* lactate dehydrogenase gene (*Pf*ldh) as previously described [26]. All reactions were run in duplicate on Applied Biosystems HT7900 Real-Time system (AppliedBiosystems, Foster City, CA, USA). Each reaction plate included *P*. *falciparum* strain 3D7 genomic DNA extracted from lab-cultured parasites as positive controls and a negative control with molecular-grade water, all in duplicate. For each plate, threshold lines were set manually and mean Ct was calculated for each amplified duplicate. Samples were considered *P*. *falciparum* positive if at least one amplification curve reached the threshold line.

### Recombinant proteins

The recombinant proteins were prepared and provided by Dr K. Tetteh (AMA-1, MSP-1_19_ and *PfSE*), Drs A. Mo and I. Hall (EBA-175RII), Dr D. Narum (MSP-3), Gennova, India (CSP) and Dr M. Thiesen (GLURP R0 and R2). Apical membrane antigen 1 (AMA-1) [27], merozoite surface protein 1–19 (MSP-1_19_) [28], erythrocyte binding antigen 175 Region II (EBA175RII) [29], Merozoite surface protein 3 (MSP-3) [30], circumsporozoite protein (CSP) (Gennova, Pune, India) [31], glutamate-rich protein fragment (GLURP-R0), GLURP-R2 [32] and *P*. *falciparum* schizonts extract (*Pf*SE) [33].

### Detection of IgG antibody levels to recombinant proteins by ELISA

IgG antibodies to each recombinant protein was assessed by ELISA as previously described [34]. Briefly, flat bottom microtiter plates (Immulon 4HBX, Thermo scientific, USA) were coated overnight at 4°C with malaria antigens adjusted at the following concentrations: AMA-1 at 0.5μg/ml, MSP-1_19_ at 0.18μg/ml, MSP-3 at 0.5μg/ml, *Pf*SE at 1μg/ml, EBA-175RII at 0.5μg/ml, GLURP R0 at 1μg/ml, GLURP R2 at 0.25μg/ml and CSP at 1μg/ml. After wash, the plates were blocked with 150μL of 1% skimmed milk powder in PBS/Tween (blocking buffer) per well for three hours at room temperature. Serum samples were added after wash at 1:1000 (except for AMA-1 diluted at 1:2000) in blocking buffer and incubated overnight at 4°C.

After incubation, horseradish peroxidase-conjugated polyclonal rabbit anti-human IgG (Dako, Glostrup, Denmark) diluted at 1:15,000 in PBS/0.05% Tween was added to each well and incubated for three hours at room temperature. The plates were developed with TMB substrate (Tebu-Bio, France) and read at 450nm with an MRX TcII reader (Dynex Technologies). All sera were tested in duplicate. Tests were repeated if duplicate optical density (OD) values for an individual serum sample varied by more than a factor of 1.5. Each plate included a three-fold dilution series (1:20 to 1:4860 final dilutions) of a Tanzanian hyperimmune standard serum pool. A titration curve was fitted to the ODs obtained for the standard serum dilutions by least squares minimisation using a three variable sigmoid model and the solver add-in in Excel (Microsoft), assuming an arbitrary value of 1000 Units/ml of antibody against each antigen in the standard pool. OD values were converted to arbitrary units/ml (AU/ml) using this fitted curve.

### Detection of IgG antibodies to lipopolysaccharide (LPS) from *Salmonella typhimurium* and *Bordetella pertussis* toxin (PT) by ELISA

ELISA detection of IgG to LPS from *Salmonella typhimurium* (STM LPS) was modified from [35] and as described in the previous section, except ELISA plates (Immulon 4HBX, Thermo scientific, USA) were coated overnight at 4°C using 100μL of coating buffer (1L PBS/1.59g Na_2_CO_3_/2.93g NaHC0_3,_ pH 9.4–9.6) per well containing STM LPS (TLRgrade, Enzo Life Sciences, NY, USA) adjusted at 5μg/ml. Following washing, plates were blocked with 200μL blocking buffer (1% skimmed milk powder in PBS) per well for one and a half hours at 37°C. Serum samples diluted at 1:100 in sample dilution buffer (1% skimmed milk powder in PBS/Tween) were added and incubated for 1 hour at 37°C. The protocol was completed as previously described in the previous section.

For PT ELISA plates (Immulon 4HBX, Thermo scientific, USA) were coated overnight at 4°C with 50μl per well of PT (NIBSC, Hertfordshire, UK) in coating buffer adjusted at 200 IU/ml. After washing, plates were blocked with 150μL blocking buffer for 1 hour at room temperature. Serum samples were diluted at 1:100 in blocking buffer and incubated for 2 hours at 37°C. After washing, 50μL of horseradish peroxidase-conjugated polyclonal rabbit anti-human IgG (Dako, Glostrup, Denmark) diluted at 1:15,000 in PBS/0.05% Tween was added to each well and incubated for three hours at room temperature. Plates were processed as described in the previous section.

### Statistical analysis

IgG antibody titers to all antigens were obtained in HEU and HUU children at different ages. Antibody titers were logarithmically transformed following which the transformed data were tested if they followed a normal distribution. The transformed data were observed not to follow normal distribution and non-parametric test were used in subsequent analysis. For each antigen, differences in the continuous antibody titers between groups at each time point were analyzed by a Wilcoxon rank-sum test. The anti-logged median titers plus interquartile ranges are presented within text. We used a Wilcoxon matched pairs signed ranks test to test whether the antibody titers to each antigen changed with time from baseline age of six months in both groups of children. Using the normalized OD values (ELISA normalized ODs) for each malaria antigen, a finite mixture model was used to define a cut-off value as previously described [36] above which the antibody response was deemed positive and below negative. The prevalence of positive antibody responses in each study group was calculated as the proportion of samples with OD above this cut-off. The breadth of antibody responses in each group at each time point was defined as number of positive antibody response to at least two or more antigens per sample. Differences in the proportions of positive antibody responses between the study groups at each time point were estimated by Chi-square and Fisher’s exact test where appropriate. Data analysis was performed using STATA (version 12) software for Mac (StataCorp LP, College Station, Texas, USA). Differences were considered to be statistically significant when the *p* value was equal to or less than 0.05.

## Results

### Participant characteristics

Both the HEU and HUU groups were comparable for both baseline and follow up characteristics (Table 1). The median birth weight was similar between HEU and HUU children (3.2Kgs vs. 3Kgs, *p* = 0.21). All mothers of the HEU children were on ART and were taking daily CTX prophylaxis during the entire follow up period. The number of children with asymptomatic malaria at each scheduled visit was low in both groups, one (3%) at 6 month, 2 (6.5%) at 12 months and 2 (6.7%) at 18 months of age for HEU children and 2 (6.5%) at 6 months, none at 12 months and 2 (7.1%) at 18 months of age for HUU children. All PCR confirmed parasitaemic children were included in the final analysis and their IgG levels are provided in S1 Table. The number of HEU and HUU children with at least one episode of clinical malaria at any time of the follow up period was determined. Three (9.1%) of the HEU children had at least one episode of clinical malaria during the follow up period compared with four (12.9%) of the HUU children.

**Table 1:** Demographic characteristics of study participants.

**Parameter** ^***a***^	Age of study participants at each visit								
	**6 months**			**12 months**			**18 months**		
	HUU	HEU	* *p*	HUU	HEU	*p*	HUU	HEU	*p*
**Demographic data**									
Number of children at each visit	31	33	-	29	31	-	28	30	-
Females (%)	65	46	-	62	42	-	64	40	-
**Physical characteristics**									
Weight (Kg)	8	8.3	*0*.*37*	9.2	9.5	*0*.*33*	10.1	10.8	*0*.*33*
Body temperature (°C)	36.8	36.7	*0*.*11*	36.5	36.4	*0*.*66*	36.4	36.2	*0*.*95*
MUAC (cm)	14	14	*0*.*88*	14	14	*0*.*79*	14	14	*0*.*82*
**Laboratory parameters**									
Mean parasitaemia	1200	7820	-	-	-		4920	1060	-
WBC count (x10^3^/μL)	10.2	9.45	*0*.*51*	11.5	12.0	*0*.*65*	9.5	8.4	*0*.*34*
Total B cells (%)	20.7	23.3	*0*.*9*	20.9	20.7	*0*.*98*	18.8	20.1	*0*.*91*
Haemoglobin (g/dL)	9.6	10.2	*0*.*17*	10.2	10.7	*0*.*51*	10.8	11.5	*0*.*13*
Platelets (x10^3^/μL)	466	369	*0*.*046*	390	434	*0*.*57*	390	390	*0*.*88*
**Malaria control measures**									
Child slept under a net (%)	86.7	93.8	-	95	95.5	-	100	100	-
If used treated net (%)	84.6	90.3	-	100	100	-	100	96.8	-

Abbreviations: P = *p* value, MUAC = mid upper arm circumference, WBC = white blood cells

^*a*^ Presented as medians

*P value obtained with Mann-Whitney U test, *p* value of <0.05 was considered significant

### Gradual increase in malaria antibody responses in HEU but not HUU following CTX prophylaxis cessation

**Fig 1:**
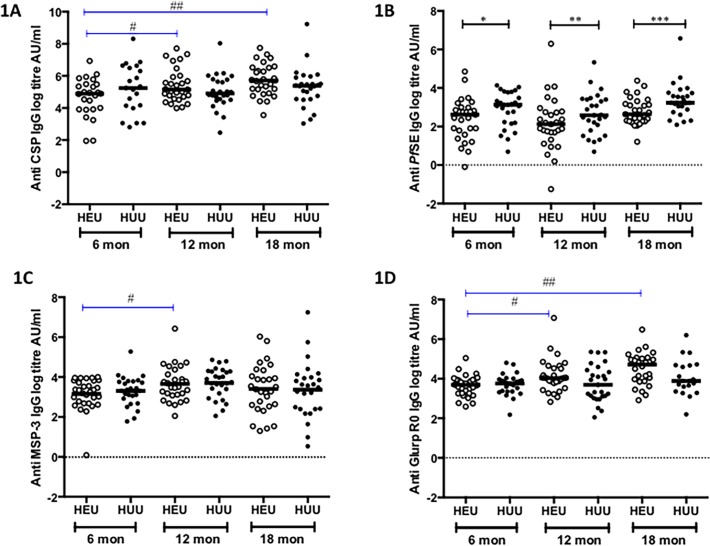
Specific IgG responses to *P*. *falciparum* antigens in HEU and HUU children at different ages. Levels of specific IgG titres were measured against CSP (**1A**), *Pf*SE (**1B**), MSP-3 (**1C**) and GLURP R0 (**1D**) in HEU and HUU at three time points, 6 months (during CTX prophylaxis), 12 months (At stopping CTX prophylaxis) and 18 months (6 months after CTX prophylaxis). X-axis represents age of children in months. Black horizontal bars represent medians. Black lines represent differences in the median titres between groups at each time point. Blue lines represent a significant change in levels of IgG titres from baseline in each group. Significant differences are indicated in asterices: **Fig. 1A** # (*p* = 0.0401), ## (*p* = 0.0018), **Fig. 1B** *(p = 0.038), **(p = 0.0012), ***(p = 0.0097), **Fig. 1C** # *(p* = 0.0463), **Fig. 1D** # *(p* = 0.0016), ## (*p* = 0.0007)

Significant variations of antibody concentrations over time were determined in both study groups. Antibody responses to CSP were measured to assess the level of infection to malaria antigens during the follow up period in both study groups [36]. IgG antibody responses against CSP in HEU children showed a steady increase with time observed from 6 to 12 months, *p* = 0.04 and 6 to 18 months, *p* = 0.0018 (Fig. 1A). However, within HUU children the antibody responses remained constant. Among HEU children, there was an increase in the IgG antibody titres from baseline against MSP-3 from 6 to 12 months, *p* = 0.0463 (Fig. 1C) and against GLURP R0 from 6 to 12 months, *p* = 0.0016 and 6 to 18 months, *p* = 0.0007 (Fig. 1D). No significant increase in the IgG antibody responses against the rest of the antigens over time was observed among HUU children (Figs. 1A-1D).

**Fig 2:**
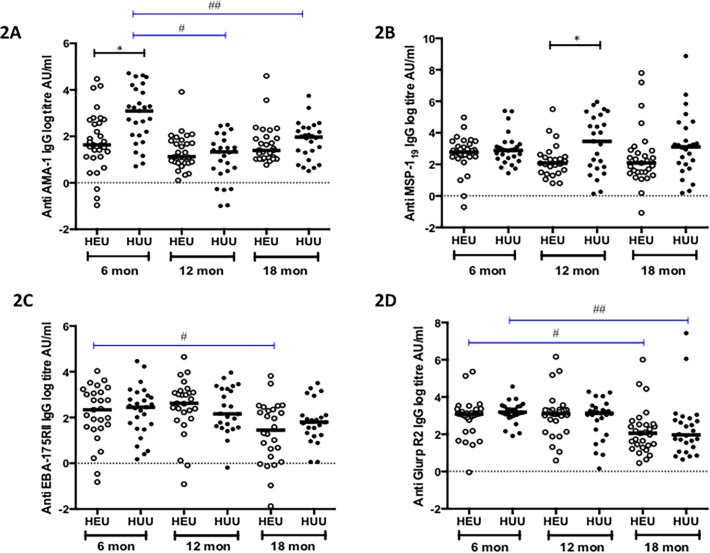
Specific IgG responses to *P*. *falciparum* antigens in HEU and HUU children at different ages. Levels of specific IgG titres were measured against AMA-1 (**2A**), MSP-1_19_ (**2B**), EBA-175RII (**2C**) and GLURP R2 (**2D**) in HEU and HUU at three time points, 6 months (during CTX prophylaxis), 12 months (At stopping CTX prophylaxis) and 18 months (6 months after CTX prophylaxis). X-axis represents age of children in months. Black horizontal bars represent medians. Black lines represent differences in the median titres between groups at each time point. Blue lines represent a significant change in levels of IgG titres from baseline in each group. Significant differences are indicated in asterices: **Fig. 2A** *(*p* = 0.0059), # (*p* < 0.0001), ## (*p* = 0.0015), **Fig. 2B** *(*p* = 0.037), **Fig. 2C** # (*p* = 0.0372), **Fig. 2D** # (*p* = 0.0107), ## (*p* = 0.0021).

However, among HEU children, there was a significant reduction in the IgG antibody titers against EBA175RII from 6 to 18 months, *p* = 0.0372 (Fig. 2C) and against GLURP R2 from 6 to 18 months, *p* = 0.0107 (Fig. 2D). Among HUU children, there was a significant reduction in the IgG antibody concentrations against AMA-1from 6 to 12 months, *p* < 0.0001 and 6 to 18 months, *p* = 0.0015 and against GLURP R2 from 6 to 18 months, *p* = 0.0021 (Fig. 2A). No significant change from baseline in the IgG titres was observed to the remaining antigens in both HEU and HUU children (Fig. 1 and 2).

### Lower magnitude of malaria antibody titers in HEU compared to HUU infants

The magnitude of the IgG antibody responses at the specific ages was then compared between the study groups. HEU children had significantly low levels of IgG antibody titres to *Pf*SE compared to HUU children at 6 months (13.7AU/ml [IQR 6.0–19.6] vs. 22.9 AU/ml [IQR 8.92–43.6] *p* = 0.0388), at 12 months (6.6 AU/ml [IQR 3.32–13.4] vs. 17.9 AU/ml [IQR 9.1–32.5] *p* = 0.0012) and at 18 months (13.9 AU/ml [IQR 9.69–23.8] vs. 25.5 AU/ml [IQR 17.9–41.8], *p* = 0.0097) (Fig. 1B). At six months of age, HEU children had significantly lower levels of IgG antibody titers to AMA-1 compared to HUU children (5.12 AU/ml [IQR 3.06–15.09] vs. 22.1 AU/ml [IQR 7.69–56.2] *p* = 0.0014) (Fig. 2A) and to MSP-1_19_ at 12 months of age compared to HUU children (7.93 AU/ml [IQR 4.45–12.0] vs. 31.8 AU/ml [IQR 6.32–142.9] *p* = 0.0416) (Fig. 2B). No other statistically significant differences in IgG antibody titers against the rest of the antigens were observed between HEU and HUU children (Fig. 1 and 2). These results suggest that the magnitude of malaria antibody titers is lower in HEU infants possibly due to lower exposure to malaria due to the effect of CTX prophylaxis or due to HIV induced impairment of antibody immunity.

### Non-malaria antigen antibody responses are maintained over time

**Fig 3:**
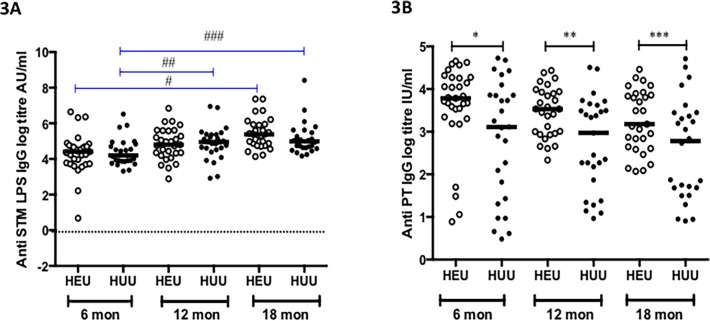
Specific IgG responses to *Salmonella Typhimurium lipopolysaccharide* and *Bordetella pertussis* toxin antigens in HEU and HUU children at different ages. Levels of specific IgG titres were measured against STM LPS (**3A**) and PT (**3B**) in HEU and HUU at three time points, 6 months (during CTX prophylaxis), 12 months (At stopping CTX prophylaxis) and 18 months (6 months after CTX prophylaxis). X-axis represents age of children in months. Black horizontal bars represent medians. Black lines represent differences in the median titres between groups at each time point. Blue lines represent a significant increase in levels of IgG titres from baseline in each group. Significant differences are indicated in asterices: **Fig. 3A** # (*p* = 0.0013), ## (*p* = 0.0263), ### (*p* = 0.0038), **Fig. 3B** *(*p* = 0.05), ** (*p* = 0.0108), *** (*p* = 0.0297)

To measure the potential confounding effect of *in-utero* exposure to HIV, we measured IgG responses to STM LPS and PT in HEU children and compared with IgG responses from HUU children. The IgG antibody titers against STM LPS were comparable between the study groups all time points (Fig. 3A). However, HEU children had significantly higher levels IgG antibody titers against PT compared to HUU children: 6 months (43.9 IU/ml [IQR 28.3–71.5] vs. 22.4 IU/ml [IQR 4.18–47.4] *p* = 0.05), 12 months (34.1 IU/ml [IQR 19.3–50.6] vs. 19.6 IU/ml [IQR 6.48–37.4], *p* = 0.011) and 18 months (23.9 IU/ml [IQR 14.1–47.4] vs. 16.2 IU/ml [IQR 5.38–31.4] *p* = 0.029) (Fig. 3B). These results suggest that the difference seen between malaria antibody responses over time between HEU and HUU are not HIV-mediated but rather reduction in malaria exposure in the HEU infants due to CTX prophylaxis.

### Limited breadth of malaria antibody responses in HEU compared to HUU infants

The breadth of antibody immune response per serum sample was described as the proportion of positive serum IgG responses to two or more blood-stage antigens. The proportion of HEU children with a positive response was significantly lower compared to HUU children at 6 months (6.45% vs. 33.33%) *p* = 0.016, but not at 12 (12.0% vs. 32%) *p* = 0.17 and 18 months of age (20.7% vs. 23.1%) *p* = 0.83. However the overall breadth of antibody response during the entire follow up was significantly lower in HEU children compared to HUU children (12.9% vs. 29.5%) *p* = 0.012) (Table 2).

**Table 2:** Proportion of HEU and HUU children with positive IgG responses to two or more *P*. *falciparum* blood stage antigens at different ages.

	Positive IgG responses to 2 or more antigens				
Age^***a***^	HEU		HUU		*p* value ^*c*^
	*n* ^*b*^	%	*n*	%	
6^*d*^	2	6.45	9	33.3	0.016*
12^*e*^	3	12.0	8	32.0	*0*.*17*
18^*f*^	6	20.7	6	23.1	*0*.*83*
Total	11	12.9	23	29.5	0.012*

^*a*^Age in Months

^*b*^ Number of positive responders to two or more antigens

^*c*^ P value determined by Fisher’s exact test for the comparison of the proportion of positive IgG responses to two or more antigens

^*d*^ Number of samples at 6 months of age: HEU (n = 31) and HUU (n = 27)

^*e*^ Number of samples at 12 months of age: HEU (n = 25) and HUU (n = 25)

^*f*^ Number of samples at 6 months of age: HEU (n = 29) and HUU (n = 26)

* Statistically significant difference at alpha level of 0.05

The prevalence of positive IgG antibody responses against AMA-1 and MSP-1_19_ were significantly reduced in HEU children compared to HUU children: AMA-1 at 6 months (16.1% vs. 51.9%, *p* = 0.048) and MSP-1_19_ at 12 months (12.0% vs. 44.0% *p* = 0.025) (Table 3). These results suggest that exposure to malaria antigens is important in the development of diverse repertoire of antibody responses against malaria antigens, and this may lead to limited breadth in malaria antibody responses in HEU infants.

**Table 3:** Prevalence of specific IgG positive response against *P*. *falciparum* blood stage antigens in HEU and HUU children at different ages.

Age^***a***^	Outcome	AMA-1			MSP-1_19_			MSP-3			*Pf*SE			GLURP R0			EBA-175 RII			GLURP R2		
		HEU	HUU	*P* ^*b*^	HEU	HUU	*P*	HEU	HUU	*P*	HEU	HUU	*P*	HEU	HUU	*P*	HEU	HUU	*P*	HEU	HUU	*P*
6^*c*^	% Pos^*f*^	16.1	51.9	*0*.*04* *	6.5	14.8	*0*.*40*	0	3.7	*0*.*47*	6.5	3.7	*1*.*0*	3.2	7.4	*0*.*59*	22.6	22.2	*1*.*0*	6.45	11.1	*0*.*66*
12^*d*^	% Pos	3.5	0	*1*.*0*	12.0	44.0	*0*.*025* *	20.7	11.1	*0*.*47*	3.45	14.8	*0*.*19*	13.8	14.8	*1*.*0*	41.8	33.3	*0*.*59*	10.3	14.8	*0*.*70*
18^*e*^	% Pos	6.9	7.7	*1*.*0*	13.8	26.9	*0*.*31*	17.2	11.5	*0*.*71*	13.8	11.5	*1*.*0*	48.3	23.1	*0*.*09*	6.9	19.2	*0*.*24*	10.3	7.7	*1*.*0*
**Total**	% Pos	8.9	20.0	*0*.*04* *	10.6	28.2	*0*.*01* *	12.4	8.8	*0*.*47*	7.87	10.0	*0*.*79*	21.3	15.0	*0*.*32*	23.6	25.0	*0*.*86*	8.9	11.3	*0*.*80*

^*a*^Age in months

^*b*^P value determined by Fisher’s exact test for the comparison of the proportion of positive IgG responses

^*c*^Number of samples at 6 months of age: HEU (n = 31) and HUU (n = 27)

^*d*^Number of samples at 12 months of age: HEU (n = 29) and HUU (n = 27)

^*e*^Number of samples at 18 months of age: HEU (n = 29) and HUU (n = 26)

^*f*^Proportion (% Pos) of positive responders to each antigen

* Statistically significant difference at alpha level of 0.05

## Discussion

We investigated the effect of daily CTX prophylaxis on the acquisition of malaria specific IgG antibodies during the first year of life in a cohort of HEU Malawian children. IgG antibody responses against seven blood stage antigens were measured in HEU children during and after stopping CTX prophylaxis and the responses were compared to aged matched HUU children.

The major strength of this study is that, matching of almost every HEU child to an HUU child from the same neighborhood meant that both cohorts were likely to be similarly exposed to malaria despite transmission varying over the course of the follow up period. The results indicate that HEU children on CTX prophylaxis have reduced specific IgG antibody responses to some malaria blood stage antigens such as recombinant merozoite surface antigens AMA-1, MSP-1_19_ and whole parasite extract *Pf*SE when compared to HUU children not receiving prophylaxis. IgG antibody responses to the other recombinant blood stage antigens i.e. MSP-3, EBA-175RII, GLURP R0 and GLURP R2 were not significantly different between HEU and HUU children during the entire follow up period. However, the study showed that overall breadth of IgG antibody response to the distinct merozoite antigens is reduced in HEU children exposed to CTX prophylaxis. This could be as a result of the delay of acquisition of IgG antibody responses to *Pf*SE, presumably caused by CTX prophylaxis related reduction in exposure to blood stage *P*. *falciparum* infection in HEU children. Ideally, HEU children not on CTX prophylaxis will have been the better controls in linking CTX prophylaxis and reduced malaria specific antibodies. Unfortunately recruitment of such a group was declared unethical by the ethics clearance body.

The use of continuous chemoprophylaxis in children and its effect on development of natural immunity to malaria has been discussed before [22,23]. In this study, despite evidence of high exposure to infection in HEU children who had higher IgG antibodies against the sporozoite antigen CSP, IgG antibodies against *Pf*SE in this group were found to be consistently lower compared to HUU children even after 6 months of stopping CTX prophylaxis. The findings are consistent with a previous report in Senegalese under-five children that showed reduced IgG antibody responses against *Pf*SE eight months after receiving a combination of sulfadoxine-pyrimethamine (SP) and artesunate, a recommended combination primarily used as malaria prophylaxis [37].

Although the previous study [37] differed with the current study, markedly the preventive therapy used (IPT with SP + artesunate versus daily CTX prophylaxis) and the age of the children (6 weeks—5 years versus 6 months—18 months), the results are in support with the current finding that suggests a specific delay in the acquisition of IgG antibodies to total parasite antigen represented by *Pf*SE in HEU children who had been exposed to CTX prophylaxis and this effect may persist for several months after stopping the prophylaxis. One possible explanation is that CTX interrupts the cumulative process in the acquisition of antibodies against *Pf*SE as a result of regular exposure to *P*. *falciparum*.

Low IgG antibody titers to MSP-1_19_were observed in HEU children at 12 months of age. Previous reports have shown that levels of antibodies to MSP-1_19_ in children depend on presence and frequent exposure to *P*. *falciparum* [12,38,39] and that these antibodies rapidly decay in the absence of exposure [40]. The results from this study showed that in addition to having high IgG antibody titers, HUU children had significantly high prevalence of positive IgG antibody responses to MSP-1_19_ (44%) compared to HEU children (12%) suggesting a lack of exposure to *P*. *falciparum* in the HEU group resulting in delayed acquisition of IgG antibodies to MSP-1_19_. However, once the protective effect of CTX was removed, HEU children were shown to rapidly acquire IgG antibodies to MSP-1_19_ suggesting a rapid acquisition of antibodies to this antigen following exposure to *P*. *falciparum*.

The observation that HEU children taking CTX prophylaxis had reduced IgG antibody responses to AMA-1 compared to HUU children at 6 months of age is consistent with results of other studies on the reduced antibody production due to lack of exposure. Low levels of IgG responses to AMA-1 were observed in African adult immigrants with no malaria residing in Spain for over two years [41] suggesting a negative effect of lack of continuous *P*. *falciparum* exposure on the levels of anti AMA-1 IgG antibodies. Despite the study being different in several aspects to the current one, the previous findings support our current observation that lack of exposure to *P*. *falciparum* as controlled by CTX prophylaxis may prevent acquisition of antibodies specific to AMA-1 in these HEU children. However, caution needs to be taken when interpreting this result from the current study since maternally transferred IgG antibodies against AMA-1 have been shown to persist for up to 12 months whilst gradually declining in Mozambican children [12]. In addition, we found a significant reduction in the IgG antibodies to AMA-1 with time in HUU children particular between 6 to 12 months, suggesting a decay of maternal IgG antibodies in these children. These results and those of another study [12] seem to suggest that it is possible that at this age, our study measured levels of maternally transferred anti AMA-1 IgG antibodies instead hence the significant difference observed between HEU and HUU children. Therefore, because of the potential confounding effect of these maternally transferred antibodies, the true effect of taking CTX on levels of IgG antibody responses against AMA-1 was difficult to ascertain.

Increasing breadth of IgG antibody specific responses has been shown to be associated with protection from clinical malaria in children [42,43]. In this study, the breadth of IgG specificity was used as a marker of cumulative exposure, which can be argued as necessary for development of protective immunity to *P*. *falciparum*, owing to the fact that children living in endemic areas are continuously exposed to numerous malaria antigens. We observed that use of CTX prophylaxis between 6 to 12 months of age was associated with reduced breadth of IgG antibodies in HEU children. However, no evidence of reduced breadth of IgG responses was observed in HEU children after 6 months of stopping CTX prophylaxis. These findings suggest that CTX may interfere with the exposure of the children’s immunity to a wide range of antigens expressed by the parasite at different stages during an infection.

*In utero* HIV exposure has been shown to be associated with lower specific antibody responses to common infections in HEU children compared with HUU children at birth [44]. To investigate whether any variation in the levels of IgG antibody responses to the blood stage malaria antigens were either due to CTX prophylaxis or *in utero* HIV exposure, IgG antibody responses to *Salmonella Typhimurium* lipopolysaccharide (STM LPS) a common infection in childhood particularly in this region [35,45–47], and EPI vaccine induced responses *to Bordetella pertussis* toxin (PT) were measured in HEU children and compared with HUU children. We found no evidence of any variation in the levels of specific IgG responses to STMLPS between HEU and HUU children. Although there have been no previous reports to support this observation, the current data suggest that reduced placental transfer of antibodies due to HIV infection does not result in significantly lower levels of IgG responses to common infection such as *Salmonella Typhimurium* in HEU children aged 6 months onwards.

Interestingly HEU children had significantly higher IgG antibody responses to PT compared to HUU children at all ages. These results are consistent with a previous study in South Africa that showed HEU children to have increased vaccine IgG responses to pertussis compared to HUU children following completion of immunization schedule [44]. A plausible explanation has been suggested in that maternal antibodies mask the vaccine epitopes thereby preventing recognition of the epitopes by the child’s developing immune system [44,48]. Taken together, these findings suggest that the observed variations in IgG antibody responses to some of the malaria antigens in HEU children were likely a result of CTX prophylaxis use rather than as a result of maternal exposure to HIV.

The study was limited in several aspects. Although the study provides clear evidence of the effect of CTX prophylaxis on the magnitude and breadth of antibody responses, it was however underpowered to evaluate the risk of clinical malaria in HEU children once the CTX intervention was stopped. Uncertainty remains on whether the so-called protective antibody levels translate to the functional immunity with responses to the same antigens appearing to be protective in some studies but not in others [49–53]. Owing to the low numbers of children with PCR confirmed parasitaemia at the scheduled visits plus the low numbers of malaria episodes, the study fails to directly determine whether the levels of IgG antibodies to the various antigens might be associated with protective immunity in these children. Therefore, IgG antibodies to these blood stage antigens appeared to be mostly markers of exposure rather than protective immunity to infection.

In conclusion, the present study confirms our hypothesis that use of CTX prophylaxis in the first year of life interferes with acquisition of natural antibody responses to blood stage malaria antigens. It has been proposed previously that anti-malaria chemoprophylaxis may interfere with the natural acquisition of malaria specific immunity. In this study, HEU children on CTX prophylaxis were shown to have reduced levels of IgG antibodies against AMA-1, MSP-1_19_ and profoundly against *P*fSE suggesting that reducing exposure early in life may delay the development of natural immunity to malaria. Secondly the breadth of specificity of the antibody responses against the malaria antigens analyzed was reduced in HEU children taking CTX prophylaxis. Given that the antibody responses observed in this study were markers of exposure, it remains to be established whether the delay in acquisition of these specific antibody responses translates into increased risk of malaria infection in HEU exposed to CTX prophylaxis.

## Supporting Information

S1 Raw Data Malaria and NTS data. (XLSX)

S1 Table IgG titres (AU/ml) to specific P. falciparum blood stage antigens in asymptomatic HEU and HUU at different ages. (DOCX)
