# Supplementary material for: The Effect of Daily Co-Trimoxazole Prophylaxis on Natural Development of Antibody-Mediated Immunity against P. falciparum Malaria Infection in HIV-Exposed Uninfected Malawian Children
Source: PLoS One. 2015 Mar 25;10(3):e0121643. doi: 10.1371/journal.pone.0121643 (PMC4373908; doi:10.1371/journal.pone.0121643)
Supplement: S1 Table — (DOCX) [file pone.0121643.s002.docx]

**Table S1**: IgG titres (AU/ml) to specific *P. falciparum* blood stage antigens in asymptomatic HEU and HUU at different ages

| **Sample** | **Age(months)** | **Group** | **AMA** | **MSP-1_19_** | **MSP 3** | ***Pf*SE** | **GLURP R0** | **EBA 175** | **GLURP R2** | **CSP** |
| --- | --- | --- | --- | --- | --- | --- | --- | --- | --- | --- |
| M022 V1 | 6 | HUU | 3.70 | 214.2 | 21.4 | 51.6 | 42.3 | 2.09 | 96.1 | 657.3 |
| M038 V1 | 6 | HUU | 2.94 | 17.5 | 27.3 | 9.29 | 28.8 | 1.71 | 32.8 | 0 |
| M057 V1 | 6 | HEU | 13.0 | 26.6 | 13.7 | 2.36 | 55.9 | 15.8 | 20.6 | 1557.1 |
| M025 V2 | 12 | HEU | 7.71 | 44.5 | 115.6 | 56.5 | 127.6 | 20.2 | 27.3 | 1418.7 |
| M031 V2 | 12 | HEU | 50.0 | 0 | 619.7 | 15.5 | 1178.6 | 25.9 | 177.2 | 1160.6 |
| M048 V3 | 18 | HUU | 9.23 | 617.2 | 1403.1 | 70.5 | 208.7 | 33.4 | 1682.8 | 1379.2 |
| M038 V3 | 18 | HUU | 25.2 | 25.1 | 32.3 | 34.4 | 61.3 | 21.5 | 8.92 | 364.2 |
| M057 V3 | 18 | HEU | 35.1 | 1339.3 | 113.4 | 43.3 | 136.4 | 45.3 | 108.9 | 728.6 |
| M061 V3 | 18 | HEU | 2.78 | 6.46 | 46.9 | 9.69 | 123.4 | 1.33 | 9.72 | 241.7 |
